# Metabolic control in type 1 diabetes patients practicing combat sports: at least two-year follow-up study

**DOI:** 10.1186/s40064-015-0919-5

**Published:** 2015-03-17

**Authors:** Teresa Benbenek-Klupa, Bartlomiej Matejko, Tomasz Klupa

**Affiliations:** DiabWay Educational Enterprise, Krakow, Poland; Department of Metabolic Diseases, Jagiellonian University Medical College, Kopernika 15, Krakow, 31-501 Poland; University Hosptal, Krakow, Poland

**Keywords:** Diabetes, Insulin pump, CSII, Combat sports

## Abstract

**Background:**

It is well recognized that physical activity should be an integral part of the management of diabetes. It remains controversial, however, whether combat sports, often preferred by young individuals type 1 diabetes mellitus (T1DM), may be performed without high risk of metabolic decompensation. The aim of this observational study was to summarize a two-year follow-up period of five young male patients with T1DM practicing combat sports under the care of a physical-activity oriented specialist diabetes outpatient clinic. Of the five patients, three mixed martial arts and two kick-boxing competitors were included in the study. To control glucose in each patient, an individual approach was used that took into consideration the type of training, the sequence of the exercises, and the relative proportion of different forms of exercise.

**Findings:**

During the follow-up, glycemic control was improved and maintained in all individuals. Neither an episode of hospitalization-requiring diabetic ketoacidosis nor severe hypoglycemia occurred in these patients during the follow-up.

**Conclusions:**

In conclusion, an individual approach for T1DM patients practicing combat sports may result in achieving and maintaining satisfactory glycemic control without increased risk of metabolic decompensation.

## Background

It is well recognized that physical activity should be an integral part of the management of diabetes. A recently published statement by the American Diabetes Association highlighted that exercise has many positive health and psychological benefits including physical fitness, weight management, enhanced insulin sensitivity, opportunities for social interactions, and building self-esteem (Chiang et al. 2014). However, intense physical activity may create new challenges for glucose control by increasing the risk of hypo- and hyperglycemia.

Carbohydrate metabolism during exercise undergoes complex regulation (Sigal et al. 2013, Francescato et al. 2004, Campaigne et al. 1987). Counterregulatory hormone secretion, and the rate of plasma glucose and glycogen utilization, varies according to the exercise type, intensity, and duration. During prolonged aerobic exercise involving continuous, rhythmic movements of large muscle groups, blood glucose levels can decrease rapidly in individuals with T1DM, thereby increasing the risk of hypoglycemia (Sigal et al. 2013, Francescato et al. 2004). On the other hand, resistance exercise involving brief, repetitive exercises with weights, may result in increase of glucose level concentration (Yardley et al. 2013a,b). Thus, combining aerobic and resistance exercise is associated with relatively stable early post-exercise glucose concentration (Yardley et al. 2013a,b). To make things more complicated, short sprints may counter an exercise mediated fall in glycemia (Bussau et al. 2006), however if performed after moderate-intensity exercise does not affect the amount of carbohydrate required to maintain euglycemia (Davey et al. 2013). High-intensity intermittent (interval) exercise, may be associated with increase in blood glucose as compared with lower intensity aerobic exercise (Sigal et al. 2013, Davey et al. 2013, Guelfi et al. 2005, Guelfi et al. 2007, Harmer et al. 2007).

Even considering these potential issues, there are a wide range of so-called “safe sports” that may be recommended for individuals with T1DM. It remains controversial, however, whether combat sports, often preferred by young individuals with T1DM, may be performed without high risk of metabolic decompensation. Combat sports require all above-mentioned forms of training (i.e., aerobic, resistance, and high-intensity intermittent activity) in variable combinations. In addition, they increase the risk of injury, often are related to high emotional stress.

## Objective

The aim of this retrospective study was to summarize the results observed during a follow-up period of at least two years for five T1DM patients practicing combat sports under the care of physical-activity oriented diabetes outpatient clinic.

## Patients and methods

Out of five patients, three were mixed martial arts (MMA) competitors and two were kick-boxing competitors. Before referral to specialist outpatient unit, all patients were treated by local diabetologists. The reason for the referral in each patient was high blood glucose variability. The major problem indicated by all individuals was glucose management following the training or competition fights.

At the beginning of the follow-up, all the patients were re-educated in the field of general principles of functional insulin therapy. With regard to glucose control, an individual approach for each patient was used, that took into consideration the type of the training (aerobic, resistance, intermittent intensive aerobic), the sequence of the exercises, and the relative proportion of different forms of exercise (Sigal et al. 2013). During the training sessions, performing resistance exercise before aerobic exercise was preferred (Sigal et al. 2013, Yardley et al. 2012). For resistance training sessions, longer, multiple sets of exercises and repetitions were advised rather than single set (15 min on the multi-gym machine) of exercise (Turner et al. 2015).

Special attention was paid to the post-combat glucose management. The suggested post-fight intervention was established on an individual basis and depended upon the fight course, and for MMA, the proportion of grappling versus striking techniques used.

In general, in the case of post-fight hyperglycemia, the correction bolus was used. However, the insulin dose was reduced by about one half of that usually given to a patient for hyperglycemia due to other factors (for example, if the patient did not have proper insulin/food dosing). In addition, in the case of patients on personal insulin pumps the insulin dose was increased by 20–30% (in relation to regular rate of basal insulin infusion) for 3–4 hours after the fight, than reduced by 20–40% for the night following the fight (to avoid hypoglycemia due to regeneration of glycogen storages).

For patients on MDI, if the post-fight hyperglycemia persisted after first correction bolus (as stated above), additional correction bolus was administered. The dose of basal insulin was reduced by 30–40% for the night following the fight.

Patients were not prescribed any medications other then insulin that could significantly affect glycemia.

For all individuals training included 2–3 hours of intense exercises per day. The number of both training and competition fights varied widely among studied patients. Patients reported average 6.7 of fights per week (range: 2/day – 3/week) and 5.4 fights per year (range: 1–9/year) for training and competition fights, respectively.

The clinical data of 5 patients practicing combat sports were compared at the end of follow-up period with the data of 16 patients, who were referred to our clinic during last 4 years, for whom data from at least two years of follow up were available, and who declared physical activity of less than hour a day with exclusion of combat sports. They were matched with respect to age, sex, HbA1c at referral. To compare two groups we used Mann–Whitney *U*-test. The Fisher’s exact test were used for categorical variables. P-values <0.05 were considered significant. Statistical analysis was carried out using Statistica PL version 10.0 (StatSoft Inc, Tulsa, OK, USA).

## Results and discussion

The clinical characteristics for all study participants are summarized in Table 1.

**Table 1:** **Clinical characteristics of study participants at the beginning and end of the follow-up period**

**Patient number**	**1**	**2**	**3**	**4**	**5**
Sex (M-male, F-female)	M	M	M	M	M
Age at the onset of follow-up (years)	18	23	34	26	31
Diabetes duration at the onset of follow-up (years)	7	13	12	9	18
Sport type	MMA	Kick-boxing	Kick-boxing	MMA	MMA
Duration of sport practice (years)	4	7	12	9	14
Diabetes treatment method (MDI/CSII)	MDI	CSII	MDI	CSII	MDI
Duration of the follow-up (months)	25	38	24	36	35
HbA1c at the beginning of the follow-up (%, mmol/mol)	8.4 (68)	8.2 (66)	8.6 (70)	7.6 (60)	7.9 (63)
HbA1c at the end of the follow-up	7.8 (62)	7.6 (60)	7.4 (57)	7.3 (56)	7.1 (54)
Mean glucose level from glucometer memory at the beginning of the follow-up (mg/dL) with standard deviation (mg/dL)	198 ± 84	189 ± 92	212 ± 89	174 ± 76	182 ± 81
Mean glucose level from glucometer memory at the end of the follow-up (mg/dL) with standard deviation (mg/dL)	179 ± 62	171 ± 68	163 ± 64	152 ± 52	139 ± 63
Number of symptomatic hypoglycemia/week at the beginning of the follow-up (4 weeks data)	8.1	10.1	8.7	6.2	12.3
Number of symptomatic hypoglycemia/week at the end of the follow-up (4 weeks data)	4.2	6.0	2.1	4.2	6.1
Number of hospitalizations due to metabolic decompensation (severe hypoglycemia or acidosis) during the 2 years prior to the beginning of follow-up	0	3	1	0	1
Number of hospitalizations due to metabolic decompensation (severe hypoglycemia or acidosis) during follow-up	0	0	0	0	0
Mean number of BG measurements/day at the beginning of follow-up	3.1	4.2	2.9	5.4	6.1
Mean number of BG measurements/day at the end of follow-up	10.2	9.1	8.1	9.3	12.4
Total daily insulin dose/kg at the beginning of follow-up (IU/kg)	0.71	0.57	0.68	0.53	0.55
Total daily insulin dose at the end of follow-up (IU/kg)	0.55	0.53	0.51	0.52	0.47
% Prandial insulin at the beginning of follow-up	46	53	58	62	66
% of total daily insulin amount given as prandial doses at the end of the follow-up	56	58	62	66	68

Grey background: data from the onset of the follow-up.

White background: data from the end of the follow-up.

Abbreviations used are: mixed martial arts (MMA), multiple daily injections (MDI), continuous subcutaneous insulin infusion (CSII), glycated hemoglobin (HbA1c), blood glucose (BG).

During the follow-up period of two years, glycemic control, as measured by glycated hemoglobin (HbA1c), was improved and maintained in all individuals. The blood glucose (BG) variability, as measured by the standard deviation of mean blood glucose level (hand blood glucometer data), was diminished. The number of symptomatic hypoglycemic episodes observed in each patient was also reduced. Of importance, there were no episodes of severe hypoglycemia, nor were there episodes of hospitalization-requiring metabolic decompensation (diabetic ketoacidosis) that occurred during the follow-up. Interestingly, the improvement of glycemic control was achieved with a lower total daily insulin dose (per kg of body weight) as the basal/prandial insulin dose was decreased in these patients.

Table 2 summarizes the comparison of selected clinical data of patients practicing combat sports with the cohort of patients who were referred to our specialist center during the same period of time (2011–2012) and who declared physical activity of less than hour a day with exclusion of combat sports. The groups were matched for age, sex, initial HbA1c. At the end of the follow up there were no differences between the groups with respect to HbA1c, however patients practicing combat sports performed more self blood glucose measurements to achieve the same level of glycemic control. The dose of insulin per kg was significantly lower in patients performing combat sports.

**Table 2:** **Comparison of selected clinical data collected during follow up of patients practicing combat sports vs. controls**

**Variables**	**At the beginning of the follow up (practicing combat sport vs others)**	**P value**	**At the end of the follow up (practicing combat sports vs others)**	**P value**
Number [N]	5 vs 16	-	5 vs 16	-
Sex [M/F]	M	-	M	-
Age [years]	26.4 ± 5.7 vs 26.1 ± 7.4	0.8357	28.4 ± 5.7 vs 28.1 ± 7.4	0.8357
CSII/MDI [N/N]	2/3 vs 12/4	0.2800	2/3 vs 12/4	0.2800
HbA1c [%]	8.1 vs 7.6	0.2824	7.4 vs 7.4	0.5071
Mean glycaemia from glucometer memory [mg/dl]	191 ± 13 vs 165 ± 45	0.1213	161 ± 14 vs 171 ± 40	0.6006
SD of glycaemia from glucometer memory [mg/dl]	84 ± 6 vs 73 ± 23	0.1662	62 ± 5 vs 73 ± 25	0.3783
Mean number of BG measurements/day [N]	4.3 ± 1.3 vs 5.1 ± 1.9	0.4068	9.8 ± 1.5 vs 4.9 ± 1.3	0.0011
Insulin dose [IU/kg]	0.6 ± 0.1 vs 0.7 ± 0.2	0.1728	0.5 ± 0.0 vs 0.7 ± 0.2	0.0082

Legend: CSII - Continuous Subcutaneous Insulin Infusion, MDI - Multiple Daily Injections, SD – Standard Deviation, BG – Blood Glucose.

We were able to achieve and maintain satisfactory metabolic control in T1DM patients practicing combat sports in our two-year follow-up study. Whilst it has been reported that any type of competition can increase the risk of metabolic decompensation in T1DM patients (Sigal et al. 2013, Murillo et al. 2010, Macknight et al. 2009, Jimenez et al. 2007), combat sports may be especially challenging in these patients. Combat sports are quite unpredictable as far as physiological responses are concerned (Chaabène et al. 2014, Bridge et al. 2009). The physiological response, including secretion of counterregulatory hormones, may depend on whether the combat is official or simulated, and whether the competitor loses or wins (Chaabène et al. 2014, Bridge et al. 2009). This is why T1DM patients practicing combat sports require an individual approach that takes the “sociophysiology of combat” into consideration.

The improvement in metabolic control in study participants may also be related to more frequent self-monitoring of blood glucose (SMBG) at the end of follow-up in comparison to the onset of the study. There is no doubt that the number and timing of SMBG should be established on the basis of individual needs of T1DM patients. For example, individuals practicing combat sports may have the highest requirement for SMBG (Chiang et al. 2014, Sigal et al. 2013, Klonoff et al. 2008).

Of interest, the majority of our patients were treated with Multiple Daily Injections (MDI) rather then Continuous Subcutaneous Insulin Infusion (CSII) via an insulin pump; despite the fact that it has previously been shown that CSII may be superior to MDI in post-exercise glycemic control (Yardley et al. 2013a,b). Individuals who refused to switch to CSII pointed to the increased risk of infusion-site related injury during combat (or need of very frequent infusion sets change that would increase the cost of CSII based management).

Finally, the improvement of glucose control was achieved with a lower insulin dose per kg of body weight. This insulin therapy optimization was probably achieved due to a lower basal/prandial insulin ratio in these patients (lower percentage total daily insulin amount given as basal doses vs prandial doses). We have shown previously that higher basal/bolus ratio may lead to impairment of the ability to perceive early symptoms of hypoglycemia. This might be especially dangerous for sport-practicing T1DM patients causing them to keep higher mean blood glucose levels (Matejko et al. 2013).

We have compared the selected clinical data of patients practicing combat sports with matched controls – patients declaring physical activity of less than hour a day with exclusion of combat sports. There were no significant differences between the groups, which may indicated that practicing combat sports itself may not lead to the deterioration of glycemic control. One should, however, underline, that our study was of retrospective and observational nature, thus drawing definite conclusions concerning the effects of combat sport on glycemic control should wait until prospective, randomized studies are performed.

The glycemic control in patients performing combat sports was also similar to that achieved in larger cohort of patients with type 1 diabetes (7.4%) treated in our Department (Matejko et al. 2015).

Undoubtedly the major limitation of our study is sample size. The small number of analyzed patients did not allow us to perform reasonable statistical analysis comparing the clinical data before and after the observation. Thus, we have presented the individual data for each study participant.

It is worth underlining, that one can not interpret the outcomes of our analysis as an indication that combat sports may lead to improvement in glycemic control in Type 1 Diabetes patients – our data simply suggest that it is possible to achieve and maintain satisfactory glycemic control despite practicing fighting sports.

Despite these limitations, to our knowledge, this is the first long-term observation concerning T1DM patients practicing combat sports.

## Conclusions

An individual approach for T1DM patients practicing combat sports may result in achieving and maintaining satisfactory glycemic control without increased risk of metabolic decompensation. It is worth emphasizing that a team with expertise in the field of physical activity in diabetes should closely manage such T1DM patients. Prospective, randomized studies are required to confirm findings based on our retrospective analysis.
